# Epidemiological and genomic determinants of tuberculosis outbreaks in First Nations communities in Canada

**DOI:** 10.1186/s12916-018-1112-9

**Published:** 2018-08-08

**Authors:** Alexander Doroshenko, Caitlin S. Pepperell, Courtney Heffernan, Mary Lou Egedahl, Tatum D. Mortimer, Tracy M. Smith, Hailey E. Bussan, Gregory J. Tyrrell, Richard Long

**Affiliations:** 1 Division of Preventive Medicine, Department of Medicine, Faculty of Medicine and Dentistry and School of Public Health, University of Alberta, Edmonton, Canada; 2 Departments of Medicine (Infectious Diseases) and Medical Microbiology & Immunology, School of Medicine and Public Health, University of Wisconsin–Madison, Madison, USA; 3 Department of Medicine, Faculty of Medicine and Dentistry and TB Program Evaluation and Research Unit, University of Alberta, Edmonton, Canada; 4 Department of Laboratory Medicine and Pathology, University of Alberta, Provincial Laboratory for Public Health, Alberta Health Services, Edmonton, Canada

**Keywords:** Tuberculosis, Outbreak, Whole-genome sequencing, First nations

## Abstract

**Background:**

In Canada, tuberculosis disproportionately affects foreign-born and First Nations populations. Within First Nations’ peoples, a high proportion of cases occur in association with outbreaks. Tuberculosis transmission in the context of outbreaks is thought to result from the convergence of several factors including characteristics of the cases, contacts, the environment, and the pathogen.

**Methods:**

We examined the epidemiological and genomic determinants of two well-characterized tuberculosis outbreaks attributed to two super-spreaders among First Nations in the province of Alberta. These outbreaks were associated with two distinct DNA fingerprints (restriction fragment-length polymorphisms or RFLPs 0.0142 and 0.0728). We compared outbreak isolates with endemic isolates not spatio-temporarily linked to outbreak cases. We extracted epidemiological variables pertaining to tuberculosis cases and contacts from individual public health records and the provincial tuberculosis registry. We conducted group analyses using parametric and non-parametric statistical tests. We carried out whole-genome sequencing and bioinformatic analysis using validated protocols.

**Results:**

We observed differences between outbreak and endemic groups in the mean number of total and child-aged contacts and the number of contacts with new positive and converted tuberculin skin tests in all group comparisons (*p* < 0.05). Differences were also detected in the proportion of cases with cavitation on a chest radiograph and the mean number of close contacts in selected group comparisons (*p* < 0.02). A phylogenetic network analysis of whole-genome sequencing data indicated that most outbreak and endemic strains were closely related to the source case for the 0.0142 fingerprint. For the 0.0728 fingerprint, the source case haplotype was circulating among endemic cases prior to the outbreak. Genetic and temporal distances were not correlated for either RFLP 0.0142 (*r*^*2*^ = − 0.05) or RFLP 0.0728 (*r*^*2*^ = 0.09) when all isolates were analyzed.

**Conclusions:**

We found no evidence that endemic strains acquired mutations resulting in their emergence in outbreak form. We conclude that the propagation of these outbreaks was likely driven by the combination of characteristics of the source cases, contacts, and the environment. The role of whole-genome sequencing in understanding mycobacterial evolution and in assisting public health authorities in conducting contact investigations and managing outbreaks is important and expected to grow in the future.

**Electronic supplementary material:**

The online version of this article (10.1186/s12916-018-1112-9) contains supplementary material, which is available to authorized users.

## Background

Tuberculosis (TB) remains a disease of major public health importance worldwide. According to the World Health Organization, there were 10.4 million incident cases of TB worldwide in 2016, most of which occur in developing countries [1]. While the incidence rate of TB in Canada is relatively low (4.6 per 100,000 in 2015), the decrease in the number of cases is slow, and minority groups, particularly foreign-born persons and indigenous peoples, are disproportionately affected [2]. This is especially true in the Canadian Prairies, including the provinces of Manitoba, Saskatchewan, and Alberta, which account for over 50% of the cases of TB among indigenous peoples in Canada [3]. Within indigenous groups, a high proportion of cases occur in connection with outbreaks [4].

Transmission of TB in the context of outbreaks is complex and is thought to result from the convergence of several factors including: (1) a highly infectious source case whose diagnosis may have been delayed, (2) a large number of susceptible contacts, and (3) an environment suitable for transmission [5]. More recently, variation among strains of *Mycobacterium tuberculosis* (MTB), which could, for example, lead to differences in transmissibility or disease progression, has been postulated as an additional explanatory factor [6]. As recently as 2012, the Public Health Agency of Canada emphasized the need to develop or enhance strategies that address TB prevention and control in high-risk settings and to conduct a TB-related program evaluation [7]. Understanding how transmission of TB occurs, as well as how infection progresses to active TB disease, particularly within high-risk settings such as outbreaks, is important for developing more effective prevention and control strategies.

DNA fingerprinting techniques (restriction fragment-length polymorphism or RFLP, mycobacterial interspersed repetitive units or MIRU, and spoligotyping) and molecular epidemiology now complement conventional epidemiology and are often used to understand TB transmission events better [8]. In recent years, whole-genome sequencing (WGS) has been increasingly used to investigate MTB outbreaks [9]. Findings from these investigations suggest that WGS may be superior to conventional genotyping for pathogen tracing and characterizing TB outbreaks at higher resolution, as well as helping to detect *super-spreaders* [10]. The discriminatory power of WGS compared to conventional genotyping methods may result in the detection of sub-clusters and the discovery of greater heterogeneity of previously defined outbreaks [10, 11]. With the decrease in the cost of WGS, its role in characterizing historical isolates of MTB is growing.

The relative contributions of pathogen versus source case, contacts, and the environment in propagating outbreaks in First Nations communities in Canada are unknown. In this study, we used conventional and molecular epidemiological tools to examine epidemiological and genomic determinants of two well-characterized outbreaks of TB among Alberta First Nations. This setting offers a unique opportunity to identify the potential role of subtle genetic variation among MTB strains in producing outbreaks, as our molecular epidemiological analysis had previously shown that the outbreaks traced to MTB RFLP types that had circulated in these communities in endemic form. The objectives of this study were (1) to explore and describe WGS characteristics and potential DNA changes between outbreak and endemic strains of MTB with the same DNA fingerprint and (2) to understand the relative contributions of pathogen versus source case, contacts, and the environment in the propagation of First Nations outbreaks. We postulated that bacterial genetic changes could potentially be associated with outbreak behavior against a stable background of circulating endemic MTB strains. Conversely, a lack of genetic change or similarity of strains from outbreak and endemic cases would suggest a greater role for the source case, contacts, or the environment in the propagation of an outbreak.

## Methods

### Settings and selection of study cases

In Alberta, all incident TB cases are reported to Alberta Health Services under the provisions of the Public Health Act [12]. All cases are recorded in the provincial TB registry (Integrated Public Health Information System or iPHIS) and all mycobacteriology testing in the province is performed in the Provincial Laboratory for Public Health (ProvLab). Since 1991, all MTB isolates from culture-positive cases have been archived in ProvLab. ProvLab also performs molecular typing on all TB isolates, generating a RFLP pattern that allows isolates to be linked to, and clustered with, other cases in the province. All outbreaks in First Nations communities are investigated by the Provincial TB Program and First Nations and Inuit Health Branch (Health Canada). In the context of outbreak management, Canadian TB standards define the initial active TB case from which the process of contact investigation begins as an *index case*, the person who was judged to be the original source of infection for secondary cases or contacts as a *source case* (who may or may not be the same as the index case), and any persons identified as being exposed to an active case of disease as *contacts.* Household contacts and those contacts with whom exposure was considered to be equivalent to that of a household setting (even if it occurred in a community setting) are considered to be close contacts and given priority for public health management [13]. Furthermore, an outbreak is defined in the Canadian TB standards as two or more identified contacts diagnosed as secondary cases of active TB in the course of contact investigation *or* two or more cases occurring within 1 year of each other and discovered to be linked, with the linkage recognized outside of contact investigation [13].

In 1992 and 1998, there were two epidemiologically defined TB outbreaks in two unrelated and spatially distinct First Nations’ communities in northern Alberta. The outbreaks were reported as such in contemporaneous government records [14, 15] and later in the peer-reviewed literature [4]. They were characterized as related to transmission from two highly infectious smear-positive, HIV-seronegative adult source cases, who had an intense cough. The outbreaks were associated with two unique RFLP types: 0.0142 and 0.0728. Each source case was associated with RFLP-specific clusters of 17 (type 0.0142) and 10 (type 0.0728) secondary cases, respectively, which were diagnosed within 12 months of the diagnosis of the presumptive source case.

From 1991 to 2013, there were also 6 and 40 endemic cases (defined as not meeting the above outbreak definition) of RFLP 0.0142 and 0.0728, respectively. Among those, one (RFLP 0.0142) and 14 (RFLP 0.0728) were smear-positive pulmonary cases. They were considered to have the potential to cause an outbreak but none of them resulted in outbreaks. The remaining 5 and 26 endemic cases of the 0.0142 and 0.0728 strains, respectively, were pulmonary smear-negative and non-pulmonary TB cases and were deemed non-infectious. Non-pulmonary cases included two cases of tuberculous lymphadenitis, one case of pleural TB, one case of central nervous system TB, and one case of disseminated TB. All isolates were susceptible to first-line anti-tuberculous medications. In total, 75 cases recorded in the TB registry were included in the study and were classified into four groups: outbreak source cases as determined by public health authorities (group 1a), secondary outbreak cases (group 1b), endemic potential source cases that did not cause an outbreak (group 2a), and endemic non-infectious cases (group 2b) (Table 1).

**Table 1:** Summary of outbreak and endemic cases (RFLP types 0.0142 and 0.0728) in First Nations communities in Alberta Canada, 1991–2013

	Outbreak cases		Endemic cases	
RFLP type	Group 1a	Group 1b	Group 2a	Group 2b
0.0142	1	17	1	5
0.0728	1	10	14	26

*RFLP* restriction fragment-length polymorphism

### Conventional epidemiological and statistical analysis

The provincial TB registry and individual public health records on outbreak and endemic cases of RFLP types 0.0142 and 0.0728 were examined retrospectively. We abstracted and derived the following epidemiological variables pertaining to characteristics of cases and contacts: age of case, presence of cavitation on chest radiograph, number of total and close contacts per case, number of child-aged contacts (<15 years old) per case, and the number of new positive tuberculin skin tests (TSTs) and TST conversions among contacts. Individuals in group 1b were secondary TB cases of individuals in group 1a and also regarded as contacts of those in group 1a. As transmission was judged to have occurred from a source case, the contacts of those in group 1b were different individuals to the contacts of those in group 1a. We also examined a place of residence of TB cases to determine whether they were on- or off-reserve at the time of diagnosis. Reserve areas are defined by the Canadian Indian Act [16]. Reserve status was determined based on individual reporting and was characterized as “yes” when a case reported a reserve as their usual place of residence or where they lived most of the time. We examined the season during which TB was diagnosed among cases, since winter in the Canadian Prairies is long and leads to increased indoor activities, potentially increasing the risk of transmission particularly in crowded living conditions. In the context of our northern Canadian climate, the season was defined as warm or cold [17]. Aggregate census data by the community of residence of cases was used to assess housing density and mean household income.

Reserve status, season, housing density, and household income were used as proxy measures of the physical and social environment in which cases lived. The distributions of continuous data variables (age; numbers of total, close, and child-age contacts; and numbers of new TST positive tests and TST conversions) were tested for normality by a Shapiro–Wilk test. For variables displaying a normal distribution, we also checked the distribution of the residuals. We conducted group comparisons separately and sequentially to determine the difference in epidemiological factors between outbreak and endemic cases. Specifically, to compare infectious cases that led to an outbreak against infectious cases that did not lead to an outbreak, we compared groups 1a and 2a. To evaluate the impact of less infectious cases on potential transmission, we included group 2b. And finally, to determine whether any secondary cases identified in group 1b could act as secondary source cases, we examined all four groups. Our three- and four-group analyses included global comparisons between all respective groups. We computed the mean, standard deviation, median, and minimum and maximum for normally distributed variables; the mean, median, minimum and maximum, and interquartile range for non-normally distributed variables; and proportions and percentages for categorical variables. We used one-way ANOVA or Kruskal–Wallis tests to examine group differences for continuous variables that were or were not normally distributed, respectively. A Fisher’s exact test was used to examine group differences for categorical variables. For group comparisons, we combined both RFLP types. We performed adjustments for multiple comparisons using a Benjamini–Hochberg procedure with a false discovery rate of 0.1 [18]. Furthermore, we separately examined group differences while excluding non-pulmonary cases from group 2b, as contact tracing may not be necessary under these circumstances. Statistical analyses were performed using STATA version 14.0 [19].

### Whole-genomic sequencing and bioinformatics

We sought every MTB isolate with each of the two described RFLP types using WGS to compare outbreak isolates to isolates not epidemiologically linked to outbreaks but with the same RFLP type. All available isolates from these two outbreaks and RFLP-matched endemic isolates were retrieved from the Alberta ProvLab archives and sent to the University of Wisconsin–Madison for WGS, which was performed on 67 isolates. WGS was not performed on eight cases (four from group 1b, one from group 2a, and three from group 2b) as seven isolates from cases reported in the TB registry were unavailable and one isolate did not yield DNA for sequencing.

#### Bacterial strains and growth conditions

MTB isolates were identified by the ProvLab by standard MTB isolation and identification assays. Isolates were stored at -70 °C until required. To perform genomic DNA sequencing, MTB strains were retrieved from storage at -70 °C and thawed. Then, 100 μl of each strain was inoculated into 10 ml of Middlebrook 7H9 broth (HiMedia) containing 0.2% w/v glycerol, 10% v/v OADC supplement (oleic acid, albumin, D-glucose, and catalase; Becton Dickinson) and 0.05% w/v Tween-80, and incubated at 37 °C with shaking for 3–5 weeks.

#### DNA extraction

Once cultures reached OD_600_ ~ 1, gDNA for WGS was isolated by following the Qiagen Genomic DNA Handbook protocol (08/2001) for bacteria using a Qiagen Genomic-tip 20/G. Briefly, cultures were transferred to 50 ml Falcon tubes containing ~35 3-mm glass beads and placed on a shaker overnight to break up clumps. Cultures were then pelleted at 3780 g for 10 min at 4 °C and re-suspended in Buffer B1. From this point, we followed the “Sample Preparation and Lysis Protocol for Bacteria” and “Protocol for Isolation of Genomic DNA from Blood, Cultured Cells, Tissue, Yeast, or Bacteria.”

#### Whole-genome sequencing

Library preparation was performed using a modified Nextera protocol as described by Baym et al. [20] with a reconditioning PCR step with fresh primers and polymerase for an additional five PCR cycles to minimize chimeras and a two-step bead-based size selection with a target fragment size of 650 bp. Libraries were submitted to the University of Wisconsin–Madison Biotechnology Center. The quality and quantity of the finished libraries were assessed using an Agilent DNA High Sensitivity chip (Agilent Technologies, Santa Clara, CA) and Qubit dsDNA HS Assay Kit, respectively. Libraries were standardized to 2 μM. For all samples, paired-end 250-bp sequencing was performed using HiSeq v1.5 SBS chemistry on an Illumina HiSeq 2500 sequencer. Images were analyzed using the Illumina Pipeline, version 1.8.2. Our raw WGS data have been deposited in the Sequence Read Archive of the National Center for Biotechnology Information (accession PRJNA390065).

#### Reference-guided assembly and variant calling

The quality of reads was checked with FastQC [21]. Reads were trimmed to remove adapters and low-quality bases using Trim Galore v 0.2.6 [22]. We mapped reads to H37Rv (NC_000962.3) using the BWA-MEM v 0.7.7 algorithm [23]. We used GATK v 2.8.1 [24] for indel realignment and variant calling. Structural variants were identified using Pindel v 0.2.5b6 [25]. We created single-nucleotide polymorphism (SNP) alignments from variant calls produced by reference-guided assembly as described above. SNPs falling in repetitive regions of the MTB genome (i.e., PE/PPE genes) and SNPs that corresponded to a gap or an ambiguous call in other isolates were removed. The alignment for RFLP types 0.0142 and 0.0728 contained 13 and 168 total SNPs, respectively, after filtering. Alignments of SNPs specific to the individual RFLP types and a VCF of all SNPs are available at FigShare (https://doi.org/10.6084/m9.figshare.5101039).

We used networks to visualize the relationships between bacterial isolates. In a network, multiple lineages can descend from a single node, and nodes may be connected in a multitude of ways without conforming to a bifurcating structure. We used the methods implemented in PopART to create median joining networks from SNP alignments [26] and to visualize genetic distances between isolates for each RFLP type.

## Results

### Descriptive epidemiology of outbreak and endemic cases

**Fig. 1:**
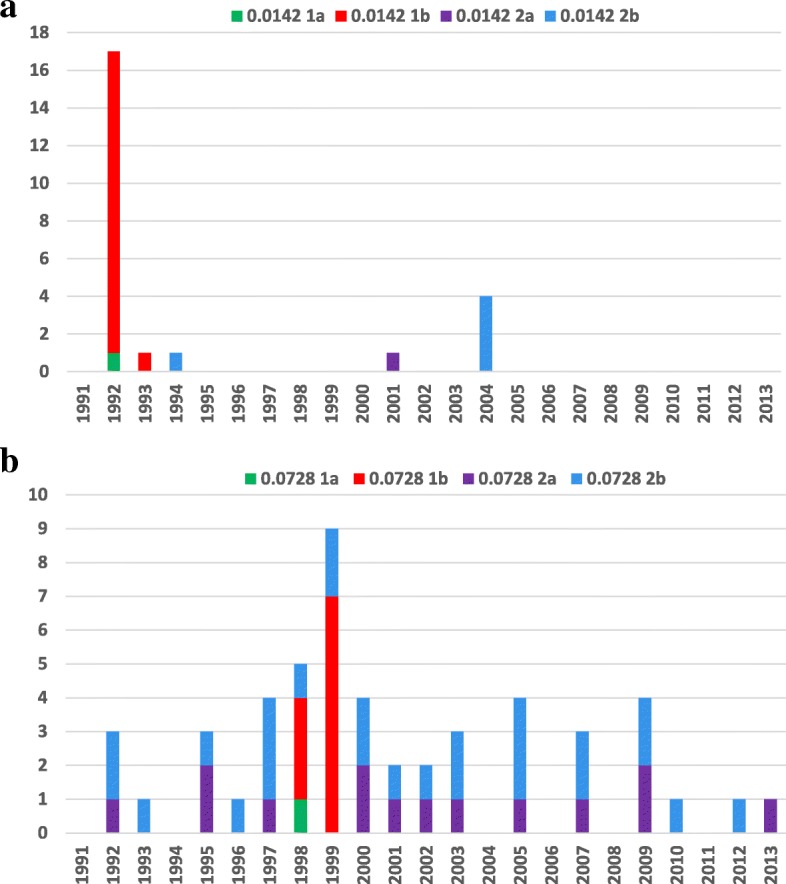
Number of outbreak and endemic cases in First Nations communities in Alberta, Canada, 1991–2013. **a** RFLP type 0.0142 and **b** RFLP type 0.0728. RFLP restriction fragment-length polymorphism

The distribution of outbreak and endemic cases of RFLP types 0.0142 and 0.0728 over time is shown in Fig. 1. For comparison, public health authorities in Alberta received 3450 notifications of TB between 1991 and 2013 and 619 of those notifications were cases among indigenous peoples (personal communication with the TB control program in Alberta). Outbreak and endemic cases belonging to the 0.0142 RFLP type occurred over a shorter interval (during 5 out of the 23 years we analyzed), while cases belonging to the 0.0728 RFLP type were more evenly spread over time (occurring in 17 out of the 23 years under observation). Most cases in the 1992 outbreak occurred during a single calendar year, while in the 1998 outbreak they occurred over two calendar years. Other than outbreak years 1992 and 1998, for which most cases were reported, there were five other years when at least four endemic cases (cases from groups 2a and 2b for both RFLP types) occurred (in 1997, 2000, 2004, 2005, and 2009). There were a greater number of 0.0728 RFLP type smear-positive cases, which could have potentially led to outbreaks compared to similar cases belonging to the 0.0142 RFLP type. Despite this apparent differential outbreak potential, we observed only one outbreak for each RFLP type.

A statistical summary of selected case, contact, and environmental attributes and group comparisons are shown in Tables 2 and 3, respectively. Among the continuous variables, only the age of cases had a normal distribution (*p* = 0.095). When comparing infectious source cases that led to outbreaks with infectious cases that did not result in outbreaks (groups 1a versus 2a), there were significant differences in the mean number of total contacts per case and the mean number of new TST positive tests among contacts (*p* < 0.05). Differences remained statistically significant when only child-age contacts and TST conversions among contacts were compared. In a three-group comparison (adding group 2b), there were also statistically significant differences in the proportion of cases with cavitation on a chest radiograph and the mean number of close contacts per case. These differences had a higher level of statistical significance when we compared all four groups (*p* < 0.001). Statistically significant differences in the age distribution of cases and in the proportions of cases living on a reserve and those who were diagnosed during the winter months were only detected in the all-group comparison (*p* < 0.007). Adjustments for multiple comparisons did not materially alter our inferences (Additional file 1: Table S1). Furthermore, excluding the five cases of non-pulmonary TB in group 2b from the analysis (in the three- and four-group comparisons) did not alter our inference about statistical group differences. Aggregate census data on community-based household income revealed there was a minimal variation in mean household income in reserve communities, ranging from CAD$ 26,048 to 29,726 per annum. The range of household income for non-reserve communities was much higher, from CAD$ 46,697 to 57,085 per annum [27]. The estimates for housing density on a reserve ranged from 1.3 to 1.5 persons per room and 2.58 persons per bedroom [27].

**Table 2:** Summary statistics of outbreak and endemic cases of tuberculosis due to RFLP types 0.0142 and 0.0728 in Alberta First Nations communities, 1991–2013

	Group 1a (*n* = 2)	Group 1b (*n* = 27)	Group 2a (*n* = 15)	Group 2b (*n* = 31)
Age, years				
Mean (standard deviation)	25 (4.2)	21.4 (15.9)	37.7 (13.4)	44.4 (21.9)
Median	25	21	44	40
Minimum to maximum	22–28	0–59	18–56	1–92
Percentage of cases with cavitation on chest radiograph	100	0	57.1^#^	3.3
Number of total contacts per case				
Mean	811.5^§^	3.2	106	18.5
Median	811.5	0	51	6
Minimum to maximum	488–1135	0–34	10–672	0–189
75% –25% interquartile range	647	1	79	9
Number of close contacts per case				
Mean	72^§^	1.9	47.7	9.4
Median	72	0	17	5
Minimum to maximum	69–75	0–18	0–465	0–80
75% – 25% interquartile range	6	0	29	10
Number of child-age† contacts per case				
Mean	27	0.7	3.9	2.2
Median	27	0	2	1
Minimum to maximum	26–28	0–8	0–23	0–17
75% – 25% interquartile range	2	0	5	3
Number of new TST positive tests among contacts				
Mean	15.5	0.2	1.8	0.3
Median	15.5	0	1	0
Minimum to maximum	13–18	0–2	0–13	0–2
75% – 25% interquartile range	5	0	2	0
Number of TST conversions among contacts				
Mean	14.5	0.1	1.9	0.3
Median	14.5	0	1	0
Minimum to maximum	12–17	0–2	0–20	0–1
75% – 25% interquartile range	5	0	1	0
Percentage of diagnoses made during cold season	100	92.5	46.7	38.7
Percentage of cases living on reserve	100	25.9	73.3	48.4

^#^Adjusted for one missing value

^†^Children were defined as being aged under 15 years

^§^An entire community was screened as part of the contact investigation in one of the outbreaks described

*RFLP* restriction fragment-length polymorphism, *TST* tuberculin skin test

**Table 3:** Group comparisons between outbreak and endemic cases of tuberculosis due to RFLP types 0.0142 and 0.0728 in Alberta First Nations communities, 1991–2013

	Two-group comparison (groups 1a and 2a) *p* values	Three-group comparison (groups 1a, 2a and 2b) *p* values	Four-group comparison (all groups) *p* values
Age of cases, years	0.2127*	0.2733*	0.0001*
Proportion of cases with cavitation on chest radiographs	0.559^¶^	0.0001^¶^	<0.0001^¶^
Mean number of total contacts per case	0.0369**	0.0001**	0.0001**
Mean number of close contacts per case	0.0522**	0.0126**	0.0001**
Mean number of child-age contacts per case	0.0239**	0.0202**	0.0004**
Mean number of new TST positive tests among contacts	0.0241**	0.0027**	0.0007**
Mean number of new TST conversions among contacts	0.0417**	0.0001**	0.0001**
Proportion of diagnoses made during cold season	0.265^¶^	0.35^¶^	<0.0001^¶^
Proportion of cases living on reserve	0.574^¶^	0.128^¶^	0.007^¶^

*RFLP* restriction fragment-length polymorphism, *TST* tuberculin skin test

*One-way ANOVA test

**Kruskal–Wallis test (adjusted for ties)

^¶^Fisher’s exact test

### Phylogenetic relationships between endemic and outbreak isolates

**Fig. 2:**
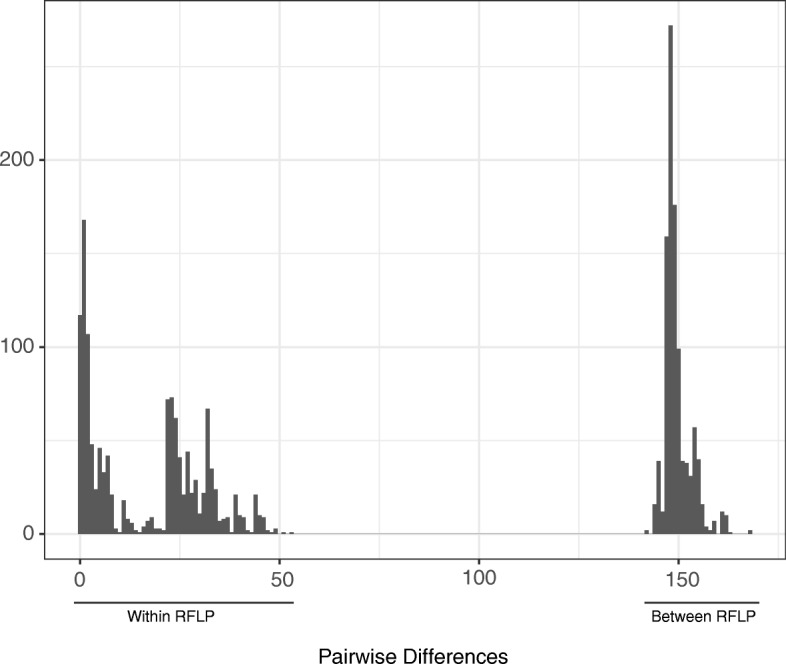
Pairwise single-nucleotide differences between isolates. SNP distances between every pair of isolates in the data set were calculated. Pairwise comparisons of isolates classified as different RFLP types have a peak at 148 differences. In comparisons among isolates with the same RFLP type, we observe a bimodal distribution of pairwise differences. This bimodal distribution reflects the structure within RFLP 0.0728, which is more genetically diverse than RFLP 0.0142. RFLP restriction fragment-length polymorphism, SNP single-nucleotide polymorphism

**Fig. 3:**
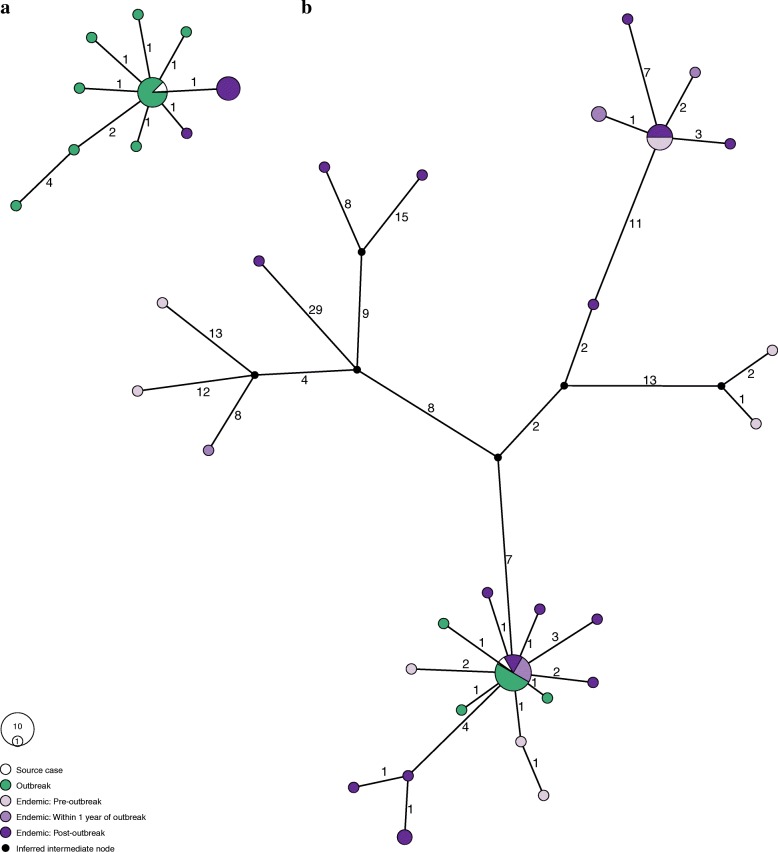
Median joining network of *Mycobacterium tuberculosis* haplotypes based on single-nucleotide polymorphisms for **a** RFLP 0.0142 and **b** RFLP 0.0728. The nodes are colored according to the outbreak and endemic classifications of the isolates. Edges are labeled according to the number of SNPs between the nodes. The size of a node is scaled based on the number of isolates with an identical sequence. The source cases for each outbreak are white (corresponding to group 1a). Secondary cases from each outbreak are green (corresponding to group 1b). Endemic cases occurring prior to the beginning of the outbreak are light purple (corresponding to group 2a). Endemic cases occurring within 1 year of outbreak cases are medium purple. Endemic cases occurring after the outbreak are dark purple (the latter two corresponding to group 2b). Black nodes are intermediate nodes inferred by the median joining algorithm. There are no isolates from endemic cases prior to the outbreak for RFLP 0.0142. Outbreak cases have 0–6 differences from the source case in RFLP 0.0142 and 0–1 differences from the source case in RFLP 0.0728. RFLP restriction fragment-length polymorphism, SNP single-nucleotide polymorphism

Isolates with the two different RFLP types had an average of 148 single-nucleotide differences between them (Fig. 2). In comparisons of isolates of the same RFLP type, there is a bimodal distribution of pairwise differences, which could represent a closely related group of strains from the outbreak versus a more diverse collection of endemic strains. However, these peaks do not correspond to comparisons between outbreak and endemic strains. SNP distances within outbreak-associated isolates and between outbreak and endemic strains are similar (Fig. 3). We compared single-nucleotide and structural variants between outbreak and endemic strains for each RFLP type and found that no variants were unique to the outbreak-associated isolates, suggesting that the outbreaks were not due to unique bacterial adaptations.

**Fig. 4:**
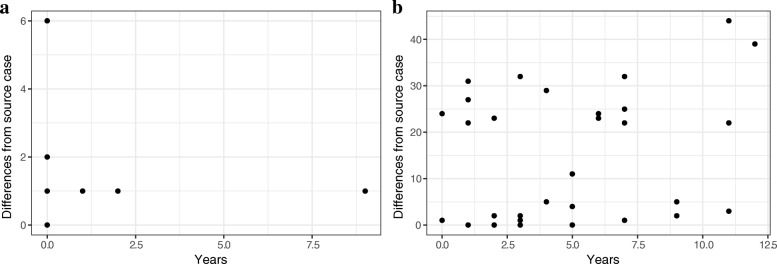
Distances from the source case for two *Mycobacterium tuberculosis* strains associated with tuberculosis outbreaks: **a** RFLP 0.0142 and **b** RFLP 0.0728. The *x*-axis shows the time interval between diagnosis in the source case and identification of a case with an identical RFLP type (temporal distance), while the *y*-axis shows the number of single-nucleotide differences between *Mycobacterium tuberculosis* isolates associated with the two cases (genetic distance). These distances are not linearly correlated. RFLP restriction fragment-length polymorphism

A network analysis of RFLP type 0.0142 strains indicated that measurable evolution occurred over the course of the outbreak (up to six SNPs), although most of the outbreak and endemic strains are closely related to the source case (one SNP distance) (Fig. 3). These analyses also demonstrated that the genomic regions examined can remain stable over long periods: strains that differed by a single SNP appeared up to 9 years after the diagnosis of the source case (Fig. 4).

Endemic strains that were closely related to the source of the type 0.0728 outbreak appeared before, during, and after the outbreak (Fig. 3). Unlike type 0.0142, type 0.0728 outbreak strains were all closely related to each other (i.e., within one SNP). Strains identical to the source appeared up to 5 years after the outbreak, and a strain that differed at three positions appeared 11 years later, indicating that the genomic fingerprint could remain stable over years for this genetic background, as well as for type 0.0142 (Fig. 4).

Two distinct lineages of endemic pre-outbreak MTB strains evolved from the source case haplotype, suggesting that the source haplotype was circulating prior to the outbreak of type 0.0728. The network also shows that genetically distant MTB isolates (e.g., 44 SNPs distant from the source case) share the same RFLP type. These genetically distant strains appeared before, during, and after the outbreak.

Phylogenetic relationships among our isolates are not well represented by a bifurcating tree (maximum likelihood trees contained 6–9 nodes with bootstrap support less than 50% dispersed throughout the trees), so instead we estimated median joining networks for both RFLP types (Fig. 3). This also precluded using root-to-top regression to estimate the substitution rate. Instead, we performed a linear regression of the temporal and genetic distances of isolates from the source case to look for evidence of a molecular clock (Fig. 4). Genetic and temporal distances did not appear to be correlated for either RFLP 0.0142 (*r*^*2*^ = − 0.05) or RFLP 0.0728 (*r*^*2*^ = 0.09) when all MTB isolates were analyzed. If a SNP threshold of 5, as described by Walker et al. [11], were used to define linked cases, the *r*^*2*^ values are 0.08 and 0.42, respectively. This indicates that the temporal signal in type 0.0728 genomic data is obscured when more distantly related MTB strains are included in the analysis. Restricting the analysis to closely related strains produces a slope of 0.36 SNPs per year.

## Discussion

In this study, we analyzed MTB genomic data combined with epidemiological data to address the question of whether TB outbreaks in First Nations communities resulted from genetic changes in endemic strains or from the characteristics of cases, contacts, or the environment. Enhanced transmissibility or virulence could emerge periodically because of ongoing evolution of endemic MTB strains, which could contribute to outbreaks of TB. MTB population genetic data from Western Canadian First Nations communities were ideally suited to investigate this hypothesis. The bacterial population consists of a small number of well-defined lineages of MTB [28, 29] and the epidemiology of TB in these communities is characterized by endemic disease punctuated by rare but explosive outbreaks or micro-epidemics [4]. Lee et al. demonstrated that a recent large outbreak in a Nunavik community involved the expansion of endemic clones of MTB, which was associated with an epidemiologic amplification, leading to a multipronged outbreak affecting >5% of the population in a small village [30]. A further in-depth analysis of WGS data is suggesting that widespread MTB infection in this region is more likely due to an environment conducive to TB transmission than a particularly well-adapted strain [31]. Lau et al. found that the rate of infection and disease among close contacts of patients with typical radiographic findings was much greater in comparison to contacts of cases with atypical chest radiographs, suggesting a primary role of case characteristics in transmitting TB [32]. Furthermore, the role of a highly infectious case in a poorly ventilated home or other restricted indoor environment was suggested as a condition for high TB infection and re-infection and consequently for outbreaks [33].

In our study, we found that there were no outbreak variants (SNPs or structural variants) common to outbreak strains but missing from endemic strains. Our results indicate that the two outbreaks likely grew out of endemic diversity: circulating endemic strains differed from the outbreak strain by 0–1 SNP for the two RFLP types. In settings with a stable endemic strain population, it may, therefore, be difficult to differentiate outbreak cases from endemic cases based on WGS alone.

Walker et al. estimated the rate of naturally occurring genetic changes identified by WGS to be 0.5 SNPs per genome per year. They also found that most epidemiologically linked cases were separated by fewer than five SNPs, suggesting a threshold of genetic changes that may signify a cluster or outbreak occurrence [11]. Bryant et al. similarly estimated a rate of 0.3 substitutions per genome per year, but they noted that the *r*^*2*^ values were low [34]. In analyzing the entire population of strains belonging to each of the RFLP types, we found little evidence of a molecular clock (*r*^*2*^ = − 0.05–0.09). However, after applying the threshold from Walker et al. to the type 0.0728 strains, we found a correlation between genetic and temporal distances as has been reported previously and a slope of 0.36 that is concordant with previous evolutionary rate estimates [34, 35]. RFLP type 0.0728 comprises MTB strains that are relatively distantly related (based on SNP distance), suggesting that the sample includes sub-populations that have diverged over long timescales (i.e., we are sampling across ancestral diversity). Larger samples and phylogeny-based inference may be needed to detect a temporal signal in the whole population. MTB rate variation related to clinical latency could also obscure temporal signals in the genomic data [36].

Our results argue against a model in which adaptation of MTB is a primary driver of TB outbreaks. The epidemiological data in our study point to outbreaks being driven by the combination of characteristics of the source cases, contacts, and the environment as postulated by Grzybowski [5]; however, we cannot definitively state which of these attributes individually played a more important role in the propagation of the outbreaks. In our two-group comparison (1a versus 2a), the difference in the proportions of cases with cavities on chest radiographs and in the mean number of close contacts did not reach statistical significance. However, this is more likely due to the smaller sample size and less balanced groups rather than being clinically insignificant. With a larger sample (three-group comparisons), these differences became statistically significant; however, cases in group 2b were considered less infectious. Finally, there were only a small number of secondary cases (i.e., cases in group 1b) who had their own contacts convert their TSTs or had a new positive TST without documented conversion. This argues against the possibility that these secondary TB cases acted as significant secondary sources. A high rate of disease progression among contacts could also result from a particularly aggressive MTB strain but, as noted above, our results do not support this. An alternative explanation is that the source cases had an intense cough and an unusually high bacterial burden and produced aerosols that delivered a large or repeated inoculum to their contacts [37]. It is also plausible that environmental conditions contributed to these outbreaks. However, census data on housing density do not show changes in association with the outbreaks and simply may not be granular enough to detect differences among households that propagated outbreaks versus those where transmission was limited. The season of TB diagnosis may be affected by the poor access to care and that other respiratory conditions are investigated before TB is considered during the winter season. A recent study by Acuna-Villaorduna et al. [38] emphasized the importance of obtaining information on proximity of exposure using simple qualitative terms. It is important that public health authorities collect reliable data on the intensity of coughing and the proximity of exposure in TB outbreak investigations.

Our study has several limitations. Molecular fingerprinting (i.e., RFLP typing) of MTB isolates in Alberta started in 1990. Therefore, we do not have molecular data on TB cases that could be related to the two RFLP types of interest in this study prior to that time. We cannot, therefore, definitively say that there were no endemic cases of RFLP type 0.0142 prior to the 1992 outbreak. The presence of endemic cases of RFLP type 0.0728 before and after the 1998 outbreak and their distribution over time suggests that endemic cases of RFLP type 0.0142 did exist prior to the 1992 outbreak and our haplotype network suggests that this was indeed the case. The retrospective nature of our study limited our ability to obtain data of sufficient completeness or granularity on all symptoms and co-morbidities among cases as well as the environment of affected communities to allow for a more comprehensive analysis of these contributions to TB outbreak propagation. Also, in small closely knit communities, all community members can be listed as contacts of a single source case, i.e., attributed to just one individual (as occurred with one of our source cases) even though the contacts can be multi-directional. Our statistical analysis of conventional epidemiological data was affected by the small number of observations in group 1a, which in turn was restricted by the number of source cases associated with the two outbreaks we studied. This limitation can potentially be overcome by studying similar outbreaks in other jurisdictions. Finally, since we used short-read technology to sequence the isolate genomes, we were unable to identify variants within repetitive regions of the genome such as PE/PPE genes, which could contain variants unique to the outbreak strains.

We found that while WGS offers improved resolution of transmission networks relative to other molecular typing techniques, our results show that distinguishing outbreak strains from an endemically circulating bacterial population can be challenging using WGS alone. It can be argued, based on our WGS findings, that our endemic strains could simply be an unrecognized part of an outbreak or secondary outbreaks. We believe, however, that the spatio-temporal differences between our outbreak and endemic strains are substantial (endemic cases were scattered among multiple northern and relatively isolated communities over a period of up to 23 years) and argue against this assertion. Moreover, outbreaks usually come to the attention of public health authorities after cases linked by conventional spatio-temporal characteristics are reported. At that time, opportunities for public health preventive measures to limit outbreak propagation (contact tracing, finding secondary cases, TST/QFT testing, and latent TB treatment) are optimal. The further in time and space cases of TB are from the epicenter of an outbreak (regardless of whether they can be linked by WGS or not), the more challenging it is to establish that transmission indeed occurred at the time of the initial event rather than as a result of remote infection.

## Conclusions

The role of WGS in understanding mycobacterial evolution and in assisting public health authorities in conducting contact investigations and managing outbreaks is important and expected to grow in the future. In our study, we did not find evidence to support the hypothesis that endemic strains of MTB acquired mutations resulting in their emergence in outbreak form. In this setting, we found that the propagation of TB outbreaks appeared to be driven by the combination of characteristics of the source cases, contacts, and possibly the environment. Preventive measures should concentrate on prompt diagnosis and effective management of cases and identification of contacts. There is a strong need to collect, prospectively and in finer detail, data relating to symptoms among cases, the environment, and socio-economic factors affecting First Nations communities.

## Additional file

Additional file 1: **Table S1.** Results of adjustment for multiple comparisons using a Benjamini–Hochberg procedure. (DOCX 15 kb)

## Abbreviations

ANOVA: Analysis of variance
CAD: Canadian dollar
iPHIS: Integrated Public Health Information System
MIRU: Mycobacterial interspersed repetitive units
MTB: *Mycobacterium tuberculosis*
ProvLab: Provincial Laboratory for Public Health
RFLP: Restriction fragment-length polymorphism
SNP: Single-nucleotide polymorphism
TB: Tuberculosis
TST: Tuberculin skin test
WGS: Whole-genome sequencing
