# Supplementary material for: Epidemiological and genomic determinants of tuberculosis outbreaks in First Nations communities in Canada
Source: BMC Med. 2018 Aug 8;16:128. doi: 10.1186/s12916-018-1112-9 (PMC6081810; doi:10.1186/s12916-018-1112-9)
Supplement: Supplementary file 1 — Table S1. Results of adjustment for multiple comparisons using a Benjamini–Hochberg procedure. (DOCX 15 kb) [file 12916_2018_1112_MOESM1_ESM.docx]

### **Supplemental Table 1. Results of multiple comparisons adjustment using Benjamini–Hochberg procedure**

False discovery rate = 0.1

| ***p-*values in ascending order** | **Benjamini-Hochberg significance** | **Benjamini-Hochberg P-value** |
| --- | --- | --- |
| 0.00001 | significant | 0.000135 |
| 0.00001 | significant | 0.000135 |
| 0.0001 | significant | 0.0003 |
| 0.0001 | significant | 0.0003 |
| 0.0001 | significant | 0.0003 |
| 0.0001 | significant | 0.0003 |
| 0.0001 | significant | 0.0003 |
| 0.0001 | significant | 0.0003 |
| 0.0001 | significant | 0.0003 |
| 0.0004 | significant | 0.00108 |
| 0.0007 | significant | 0.001718182 |
| 0.0027 | significant | 0.006075 |
| 0.007 | significant | 0.014538462 |
| 0.0126 | significant | 0.0243 |
| 0.0202 | significant | 0.03636 |
| 0.0239 | significant | 0.038276471 |
| 0.0241 | significant | 0.038276471 |
| 0.0369 | significant | 0.05535 |
| 0.0417 | significant | 0.059257895 |
| 0.0522 | significant | 0.07047 |
| 0.128 | not significant | 0.164571429 |
| 0.2127 | not significant | 0.261040909 |
| 0.265 | not significant | 0.3074625 |
| 0.2733 | not significant | 0.3074625 |
| 0.35 | not significant | 0.378 |
| 0.559 | not significant | 0.574 |
| 0.574 | not significant | 0.574 |
